# Mercy Hospital

**Published:** 1872-11-15

**Authors:** J. H. Hollister


### MERCY HOSPITAL.

SERVICE OF PROF. J. H. HOLLISTER.

*Catarrhal Inflammations, with Clinical Remarks upon a Case of Chronic Laryngitis.*

GENTLEMEN: — You will have observed, in passing through the wards of the hospital, that a large number of cases have presented themselves, in which the patients are suffering from what are familiarly known as *malarial diseases.* Among them we find, in different degrees of intensity, and in different stages of progress, the usual forms of intermittent, remittent, and bilious affections. But as the season advances, we shall note a marked change in this respect. We shall, indeed, have serious complications to treat, and some of them of grave character, as the *sequalæ* of these diseases; but the fresh and typical forms of malarial diseases will soon cease to present themselves for treatment for the balance of the current year.

Two other forms of disease will now come more prominently to your notice, and require a large share of your attention. They are incident to the season of the year upon which we now enter, and such is their importance, such, often, their gravity, that in the present course of instruction, they will receive at our hands very critical attention. The diseases to which I refer are the various manifestations of catarrhal inflammation and rheumatism. And as you will be called to study a variety of catarrhal affections, as different patients are presented, I wish to premise a few general remarks, upon the causes, pathology, and treatment of catarrh in general.

Catarrhal inflammation involves only mucous membranes; but wheresoever these are found, there the disease may be manifested. It consists in a *hyperœmia* of that structure, rendering it swollen and soft, and increasing very greatly the secretions from the free surface, of a mucous, viscid in character and rich in corpuscles.

The persons most liable to this disease are sometimes plethoric and of full habit — others of anæmic condition, and predisposed to local inflammation; the first most frequently being affected in the respiratory tract, the latter in the intestinal or vaginal membranes. Persons with thin and delicate skin, and inclined to perspire freely, are most liable to be affected.

There is a very marked tendency to the re-development of catarrhal diseases in parts that have been formerly affected; and their repetitions may be inaugurated by causes so slight as to render the parts almost continually the subjects of disease; so that, when treated successfully, and the patient seems cured, a repetition may bring the agravations which attended former attacks, much to the discouragement of both patient and physician.

The *causes* which produce catarrhal inflammation are various, and they need to be accurately recognized before we can treat them understandingly. We have many cases of catarrh arising from venous obstruction, preventing the flow of blood *from* the membrane. For instance, the pressure of the gravid uterus may so impede the circulation as to produce severe vaginal catarrh. Portal congestion may, and often does, produce severe intestinal catarrh; and any impediment to the return of blood from the lungs may produce the very marked development of pulmonary catarrh.

The most frequent causes of catarrh, however, are such as ordinarily induce local inflammation, such as the presence and irritation of foreign substances, as from the inhalation of dust, gases, and irritating vapors. Another fruitful source of catarrh is the subjection of the body to sudden and severe changes of temperature, either by undue exposure, or by untimely changes of clothing. Some of the eruptive diseases develop serious catarrhal affections; as in the case of measles. So, too, excessive use or exercise of parts often produces the disease; as from shouting, singing, or speaking in the open air. This affection is also often propagated from diseased parts to healthy ones in close proximity; as when, by excessive use of alcohol, the fauces are inflamed, we often have laryngitis in extreme severity: witness the hoarse cough and altered voice, and the rough breathing of the confirmed debauchee.

I have said that catarrh was dependent upon a hyperæmic condition of the mucous membrane, when, as in the case before us, the larynx is involved. To get an accurate conception of the patient's condition, it is quite essential that we inspect the parts by the aid of the *laryngiscope*, the instrument which I now have in hand, and to the method of using which, I now invite your attention. * * * The importance of thus examining the tissues during the life-time of the patient, arises from the fact that such is the elasticity of the tissues involved, that they are emptied of blood almost as soon as the circulation ceases, and fail almost entirely to represent that engorgement of the parts which is always present, while the circulation is yet active. If we inspect the tissues in cases of extreme severity, and which terminated suddenly, we shall find the membrane studded with spots of ecchymosis, giving it a mottled appearance; and this will be permanent, as it is produced by extravasation of blood.

Parts which are frequently subjected to catarrhal inflammation, have also marked involvement of the sub-mucous tissue. They become permanently thickened and hardened; and so firm may be the deposit, that re-absorption is accomplished very slowly, at least. The deposit has resemblance of condensed connective tissue, and partakes in a measure of the color of the ecchymosed patches. In fact, it is as really *hypertrophy* of that tissue, as may occur in muscular, or any other.

Usually, catarrhal inflammations must be acute and extensive, to produce severe constitutional disturbance; and so long as only local disease is developed, we shall hardly ever anticipate fatal result. Extensive acute pulmonary catarrh may perhaps seem an exception to this rule.

*Treatment:* We are to seek, first, the prevention of disease. To this end, patients should be so much accustomed to the ordinary temperatures, and to the fresh air, as to gain a tolerance of ordinary exposure. Clothing should only meet the absolute needs of *comfort*, and must vary with the conditions of patients; above all, sudden changes of clothing, whereby extreme variances of protection are effected, are *ever and always* to be avoided. The different parts of the body should be so clothed as to afford uniform protection. Mufflers upon the necks are often supplemented by bare knees — but this indicates a want of brains somewhere. Many little patients suffer by undue exposure in the night-hours, from being able, with a single throw, to bare themselves of all clothing. Another extreme may easily be reached by dressing *too warmly*, and thus by inducing free perspiration, predisposing to "colds."

When the disease can be traced to specific exposures, or causes, these, whenever practicable, should be promptly removed or avoided; as in laryngeal catarrh, rest from singing, speaking, etc., may be all that is needed to afford relief.

In cases of recent acute attack, whatever tends to equalize the circulation, and especially to establish free and general perspiration, will tend to mitigate the symptoms.

The Turkish-bath, warm-baths — various processes and appliances by which free perspiration is induced, are very efficacious.

Opium in some instances, especially associated with ipecac, as in Dover's powder, is given with benefit. Caution is always to be used in administering this remedy to children.

Pulmonary catarrhs, in chronic form, are often relieved by the judicious use of stimulating expectorants, the excessive engorgement being relieved by the increased mucous secretion. Other membranes are often benefitted by the application of stimulation to their surfaces, as by lotions, and injections, by which parts too relaxed are stimulated to a normal tonicity. In all those cases where the trouble is dependent upon constitutional debility, very little benefit can be derived from topical application, till the general condition of the patient has been improved.

Gentlemen: With these brief remarks upon the subject of catarrh in general, I bring to your notice, in the patient before us, a case of *chronic laryngitis.* We have here no constitutional disturbance. The circulation and the secretions are nearly natural. The patient suffers little pain, and his strength is sufficient. But you note his labored inspirations — his inability to speak above a whisper — and his extremely hoarse cough. With auscultation and percussion we note the absence of pulmonary complication. We find by examination that the disease is *local*, confined to the larynx. The case is clearly inflammation of the laryngeal mucous membrane, involving the glottis and vocal cords. The history of the patient shows that this is not the first attack. He is predisposed, upon exposure, to laryngitis. By occupation a sailor, in the recent stress of weather he met just that exposure calculated to develop the disease. The affection has passed through its acute stage without febrile disturbance; he being affected with *pain* upon deglutition; a severe cough; aphonia, and extreme dyspnœa.

It remains, now, to treat the secondary symptoms of laryngitis. If there were ulceration, we should seek to hasten the healing by stimulating applications, as by the throwing of powders upon the affected parts, by the use of small tubes charged with medicines, and expelled by the force of air upon the affected parts, or by the application of liquid stimulants, applied by means of the probang, as recommended by Dr. Greene; or, as later and more effectually, by the inhalation of atomized liquids, in the form of spray. But we have here no evidence of ulceration. Again, if there were evidence of syphilitic taint, we might be required to address ourselves at once to constitutional treatment; but happily we have none of this. We are left, therefore, to the treatment, of a simple, uncomplicated case of chronic laryngitis.

We have here to wait the absorption of effused plastis lymph by which the sub-mucous tissue has been thickened and hardened. We are to favor this by keeping the patient *warm*, and free from sudden changes.

The vocal organs are to be kept absolutely at rest. Opium, in the form of Dover's powder, will be given at bed-time to control the cough, and the patient instructed to abstain from coughing to the utmost extent; as his will has much to do in ensuring quietude of the parts.

A pill of hydrarg. prot. iodide, one-fourth gr. will also be given each night at bed-time. A mixture of equal parts of syrupi-scillæ comp. and syrupi-glycyrrhiza, say of each ℥ ii., to which will be added of ammoniæ mur. ℥ ss., and of tinct. sanguinaria canadensis ℥ ss. Of this mixture we direct a teaspoonful three times a day, with a view to restore the function of secretion now suspended. We direct that the surface of the neck over the larynx be irritated by dressings of croton oil, and, daily, the inhalation of the vapor of water, containing five grs. of crystal carbolic acid to the ounce, by means of the steam atomizer.

We shall anticipate in a few days the re-establishment of the normal mucous secretion, and the disappearance of cough and dyspnœa, as absorption of the effused lymph is accomplished, and with this the natural use again of the vocal organs.

